# Claudin 4 Is Differentially Expressed between Ovarian Cancer Subtypes and Plays a Role in Spheroid Formation

**DOI:** 10.3390/ijms12021334

**Published:** 2011-02-22

**Authors:** Kristin L. M. Boylan, Benjamin Misemer, Melissa S. DeRycke, John D. Andersen, Katherine M. Harrington, Steve E. Kalloger, C. Blake Gilks, Stefan E. Pambuccian, Amy P. N. Skubitz

**Affiliations:** 1 Department of Laboratory Medicine and Pathology, University of Minnesota, Minneapolis, MN 55455, USA; E-Mails: boyla002@umn.edu (K.L.M.B.); benjamin.misemer@gmail.com (B.M.); deryc004@umn.edu (M.S.D.); ande0555@umn.edu (J.D.A.); harr0750@umn.edu (K.M.H.); pambu001@umn.edu (S.E.P.); 2 Cheryl Brown Ovarian Cancer Outcomes Unit, British Columbia Cancer Agency, Vancouver, Canada; E-Mails: skalloger@mac.com (S.E.K.); Blake.Gilks@vch.ca (C.B.G.)

**Keywords:** ovarian cancer, claudin 4, biomarker, spheroid, ascites

## Abstract

Claudin 4 is a cellular adhesion molecule that is frequently overexpressed in ovarian cancer and other epithelial cancers. In this study, we sought to determine whether the expression of claudin 4 is associated with outcome in ovarian cancer patients and may be involved in tumor progression. We examined claudin 4 expression in ovarian cancer tissues and cell lines, as well as by immunohistochemical staining of tissue microarrays (TMAs; n = 500), spheroids present in patients’ ascites, and spheroids formed *in vitro*. Claudin 4 was expressed in nearly 70% of the ovarian cancer tissues examined and was differentially expressed across ovarian cancer subtypes, with the lowest expression in clear cell subtype. No association was found between claudin 4 expression and disease-specific survival in any subtype. Claudin 4 expression was also observed in multicellular spheroids obtained from patients’ ascites. Using an *in vitro* spheroid formation assay, we found that NIH:OVCAR5 cells treated with shRNA against claudin 4 required a longer time to form compact spheroids compared to control NIH:OVCAR5 cells that expressed high levels of claudin 4. The inability of the NIH:OVCAR5 cells treated with claudin 4 shRNA to form compact spheroids was verified by FITC-dextran exclusion. These results demonstrate a role for claudin 4 and tight junctions in spheroid formation and integrity.

## 1. Introduction

Ovarian cancer is the most lethal gynecological malignancy, resulting in approximately 125,000 deaths yearly, worldwide [1]. Due to the paucity of specific symptoms and the lack of an effective screening method, the majority of ovarian cancers are diagnosed at late stages of malignancy, after the tumor has spread beyond the ovary [2]. Although initial response to treatment (surgery and chemotherapy) is favorable, most patients will relapse with tumors that are chemoresistant and ultimately die of their disease.

In contrast to other solid tumors, the most common method for ovarian cancer metastasis is direct peritoneal spread. Tumor cells slough off the ovary into the peritoneal fluid where they are disseminated throughout the abdominal cavity, and subsequently attach to the mesothelial cell lining and invade, forming metastatic outgrowths [3]. Late stage cancers are frequently associated with ascites, and tumor cells can be shed into the ascites fluid either as single cells or multicellular aggregates called spheroids. Spheroids suspended in the ascites fluid were previously thought to be quiescent. However, we and others have shown that ovarian cancer spheroids are a source of tumor invasion and metastasis [4–8]. Spheroids are also chemoresistant [9,10], implicating spheroids as a factor in disease persistence or recurrence.

In an effort to find novel biomarkers for ovarian cancer using gene expression profiling, we and others [11–15] have identified claudin 4 as a gene that is highly overexpressed in ovarian cancer, and thus may contribute to tumor formation and metastasis. Claudins are a family of cellular adhesion molecules that are components of tight junctions, which play important roles in cell polarity, paracellular transport, and the formation of epithelial cell sheets which serve as a barrier in tissues. Claudin expression in normal cells is tissue specific, and altered claudin expression has been identified in multiple cancer types [16,17]. Consistent with the idea that tumor formation is associated with tight junction disruption, downregulation of claudin family members has been reported in some cancers, and is associated with a poor prognosis or metastatic disease [18–27]. In contrast, claudin expression may also be elevated in different types of cancers and associated with a metastatic phenotype [28–33].

In this study, we sought to validate the overexpression of claudin 4 that we previously observed in ovarian cancer tissues in our gene microarray experiments [12]. We have evaluated the levels of claudin 4 RNA and protein in ovarian cancer tissues and cell lines using RT-PCR, qRT-PCR, Western immunoblotting, and immunohistochemistry, in order to assess the potential of claudin 4 as an ovarian cancer biomarker. We also sought to determine whether claudin 4 overexpression could be correlated with relevant clinical outcomes using tissue microarrays comprised of 500 cases of clinically annotated ovarian cancer. Finally, because of its role in intercellular adhesion, and the importance of ovarian cancer spheroids in ovarian cancer metastasis and chemoresistance, we examined the expression of claudin 4 in ovarian cancer spheroids and its potential role in spheroid formation.

## 2. Results and Discussion

### 2.1. Claudin 4 RNA Is Overexpressed in Ovarian Cancer

Our previous analysis of gene expression in human tissues identified claudin 4 as one of 66 genes that were upregulated in serous ovarian cancer relative to normal ovaries and over 300 other normal and diseased tissues [12]. Our initial validation identified claudin 4 as one of three genes that best distinguished between ovarian carcinoma and normal ovary tissues [12]. To further validate our gene expression studies, we examined the expression of claudin 4 RNA in ovary tissues and cell lines. Consistent with our microarray data, RT-PCR showed 5/5 serous ovarian cancer tumor tissue samples tested were positive for claudin 4 compared to only 2/6 normal ovary samples (data not shown). Other groups have also identified claudin 4 RNA upregulation in ovarian cancer tissues [11,13–15] as well as pancreatic, prostate, and squamous cell carcinomas [29,31,34–36].

**Figure 1.**
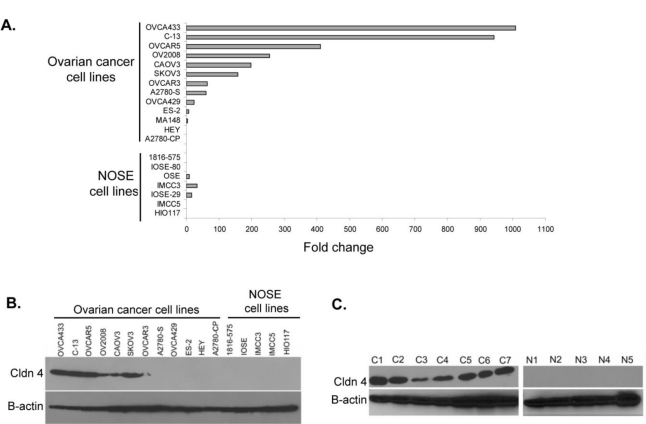
**Claudin 4 RNA and protein expression in ovarian cancer tissues and cell lines**. (**A**) Claudin 4 RNA expression in 13 ovarian cancer cell lines and 7 NOSE cell lines as determined by qRT-PCR. Expression values shown as fold change over the lowest expressing cell line (1816–575), and are the average of two experiments (see Experimental section). (**B**) Claudin 4 protein expression was determined by Western immunoblot analysis of ovarian cancer and NOSE cell lines (50 μg protein/lane). β-actin serves as a loading control. (**C**) Claudin 4 protein expression was determined by Western immunoblot analysis of 7 primary stage III/IV serous ovarian cancer tissues (C1–C7) and 5 normal ovaries (N1–N5) (50 μg protein/lane). β-actin, loading control.

Because surface epithelial cells comprise only a minor fraction of the normal ovary, we examined the expression of claudin 4 in ovarian cancer and immortalized normal ovarian surface epithelial (NOSE) cell lines. We also used ovarian cancer cell lines as a more pure population of tumor cells, without contaminating stroma and other cell types. Similar to what others have reported [32,37], we found the level of claudin 4 RNA in ovarian cancer cell lines was varied. By qRT-PCR, we found the cell lines OVCA433, C-13, OVCAR5, OV2008, CAOV3, and SKOV3 express high levels of claudin 4 mRNA, while the cell lines OVCA 429, ES-2, MA148, HEY, and A2780-CP (as well as all of the NOSE cell lines) were found to express low levels of claudin 4 mRNA (Figure 1A). The variable expression of claudin 4 in ovarian cancer cell lines suggests that claudin 4 expression may be associated with functional characteristics of the cell lines, such as proliferation rate or aggressive behavior. In other studies, the expression of claudin 4 in ovarian cancer cell lines has been shown to increase cell migration and invasion [28].

### 2.2. Claudin 4 Protein Is Overexpressed in Ovarian Cancer Cell Lines and Tissues

We next examined claudin 4 protein expression by Western immunoblot analysis in both cell lines and tissues. Claudin 4 protein was detected in the six ovarian cancer cell lines that expressed high levels of claudin 4 RNA (OVCA433, C-13, OVCAR5, OV2008, CAOV3, and SKOV3), whereas ovarian cancer cell lines with low levels of claudin 4 RNA expression and all of the NOSE cell lines were negative for claudin 4 protein by Western blot (Figure 1B). These results, coupled with the qRT-PCR data, led us to select the NIH:OVCAR5 and MA148 cell lines for subsequent experiments in this study. We observed that all seven primary tumors from women with stage III/IV serous ovarian cancer tested were positive for claudin 4 protein expression (Figure 1C). Although some claudin 4 transcripts were detected in normal ovaries by RT-PCR, by Western blotting, none of the five normal ovary tissues tested were positive for expression of claudin 4 protein (Figure 1C). These results are supported by other recent studies in which claudin 4 protein expression was demonstrated in lysates from ovarian cancer cell lines, but not in cultures of NOSE cells [32,37,38]. One caveat to this is the emerging concept that some ovarian cancers, in particular serous subtype tumors, arise from the fimbria of the fallopian tubes [39], which could limit the validity of normal ovaries for comparison of gene expression in these and other experiments [11,13–15,32,37,38].

### 2.3. Claudin 4 Protein Expression in Ovarian Cancer Tissues

**Figure 2.**
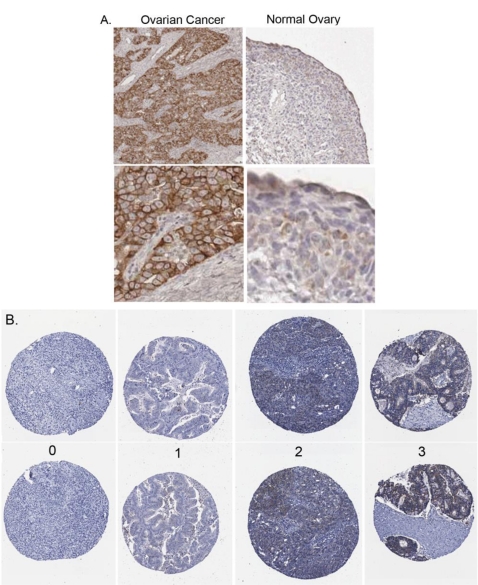
**Claudin 4 immunohistochemical staining of FFPE tissues**. (**A**) Representative claudin 4 staining of whole mount sections of serous ovarian cancer and normal ovary. Top, 200× magnification; bottom, enlargement to show detail. (**B**) Examples of claudin 4 staining and scoring for TMA samples: 0, no cancer cells staining; +1, <10% of cancer cells staining; +2, 10–50% of cancer cells staining; +3, >50% of cancer cells staining. In some cases, positive scores (1, 2, and 3) were binarized for analysis.

In previous immunohistochemical (IHC) studies, we observed that claudin 4 staining was localized to the cell membrane of frozen sections from 15 serous ovarian cancer primary tumors, and 15 serous ovarian cancer tumors metastatic to the omentum; no claudin 4 staining was observed in the surface epithelial cells of the 15 normal ovaries that were examined [12]. These earlier studies used fresh, snap-frozen tissues that were embedded in OCT, so that the antigens would not be destroyed by fixatives, increasing the likelihood that the antibodies would recognize the antigens in the tissues. In the current study, we initially performed IHC staining using a test set of 58 formalin-fixed paraffin-embedded (FFPE) tissue blocks including 21 FFPE cases of normal ovaries with intact surface epithelial cells. The FFPE tissue sections were used in order to determine if formalin fixation would interfere with the detection of claudin 4, and to optimize the staining methodology for subsequent FFPE tissue microarrays. In the test set of FFPE tissues, we observed that staining for claudin 4 in ovarian cancer cells was localized to the cancer cell membranes with some cytoplasmic blush (Figure 2A). Overall, the ovarian cancer tumor cells, but not stroma, had a high percentage of claudin 4 staining in the individual sections. Normal ovarian surface epithelium was either negative or had a slight blush of staining present. Claudin 4 staining was observed in 64% of the serous ovarian cancer tissues (21/33), 75% of the clear cell ovarian cancer tissues (3/4), and only 19% of the normal ovary tissues (4/21). The successful optimization of IHC staining for FFPE tissues and the results showing increased levels of claudin 4 expression in serous ovarian cancer tissues compared to normal ovaries, not only validated our previous results [12], but led us to examine claudin 4 expression in a much larger cohort of patients.

### 2.4. Claudin 4 Protein Is Differentially Expressed between Subtypes of Ovarian Cancer

**Figure 3.**
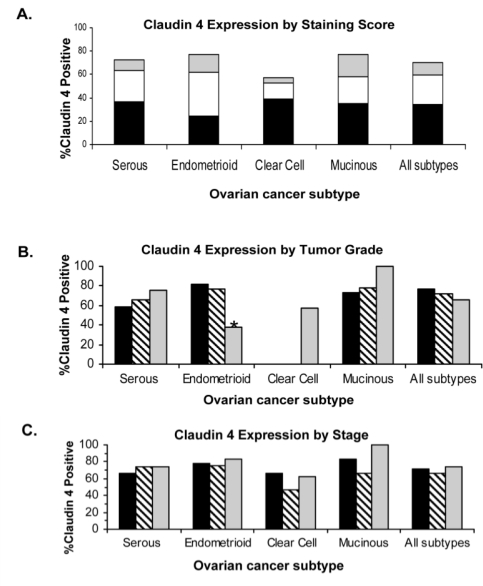
**Claudin 4 expression in ovarian cancer subtypes in tissue microarrays**. (**A**) Claudin 4 is differentially expressed in ovarian cancer subtypes (p = 0.0026). Percent of TMA cases positive for claudin 4 staining in ovarian cancer subtypes. Staining score: black, score +1 (<10% of cancer cells staining); white, score +2 (10–50% of cancer cells staining); gray, score +3 (>50% of cancer cells staining). (**B**) Claudin 4 expression by Silverberg Grade. Percent of TMA cases positive for claudin 4 by Silverberg Grade (grade 1 is black; grade 2 is diagonal stripe; grade 3 is gray). Positive scores (1, 2, and 3) were binarized. Claudin 4 expression is significantly lower in high grade endometrioid ovarian cancer (p = 0.0178). (**C**) Percent of TMA cases positive for claudin 4 by stage (stage I is black; stage II is diagonal stripe; stage III is gray). Positive scores (1, 2, and 3) were binarized. Claudin 4 expression was not significantly different between the stages.

To determine whether other subtypes of ovarian cancer would also show increased levels of expression and if claudin 4 expression in ovarian cancer was associated with outcome or other clinical parameters, we performed immunohistochemical staining of claudin 4 on tissue microarrays (TMA). The TMAs encompassed 500 cases of epithelial ovarian cancer of different subtypes (serous, mucinous, endometrioid and clear cell; Table 1; Figure 2B); each tissue was associated with patient clinical data, including up to 20 years of follow-up [40]. Overall, claudin 4 expression was observed in 69.9% of ovarian cancer patients, with differential expression observed between the different ovarian cancer subtypes (p = 0.0026; Figure 3A), due to a lower percentage of cells stained in clear cell tumors. The highest percentage of expression was observed in the endometrioid and mucinous subtypes (both 77.4% positive), compared to serous (72.17% positive), and clear cell (57.58% positive) subtypes. These results extend the previous analysis of claudin 4 expression in ovarian cancer subtypes [32,37,38,41], which are generally in agreement with our data. In these prior studies, claudin 4 expression was elevated in the majority of cases of epithelial ovarian cancer, with approximately 70% of serous ovarian cancers staining positively for claudin 4 [32,37,38,41]. However, in contrast to our results showing that the endometrioid and mucinous subtypes of ovarian cancer had the highest levels of claudin 4 expression, Litkouhi *et al.* found the highest percentage of claudin 4 staining in endometrioid and clear cell subtypes, although with a much smaller sample size [37].

Claudin 4 expression was also analyzed in each subtype by Silverberg grade and stage (Figures 3B and 3C). In the endometrioid subtype, claudin 4 was differentially expressed by Silverberg grade (p = 0.0178), and was inversely associated with grade; 81.48% of Grade 1 tumors were claudin 4 positive, 77.14% of Grade 2 tumors were claudin 4 positive, and 37.5% of Grade 3 tumors were claudin 4 positive. In contrast, previous studies that examined primarily serous tumors suggested that claudin 4 expression was increased in undifferentiated tumors [37,38]; however, those studies included only a small number of non-serous subtype tumors. No other associations between claudin 4 expression and stage or Silverberg grade were observed.

**Figure 4.**
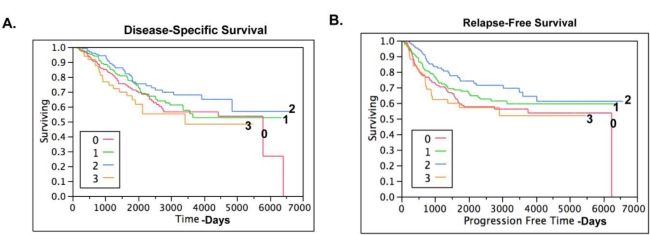
(**A**) Kaplan-Meyer survival curve of TMA data for 500 ovarian cancer patients showing disease-specific survival measured in days. (**B**) Kaplan-Meyer survival curve of TMA data for 500 ovarian cancer patients showing relapse-free survival measured in days. Red, staining score 0; green, staining score +1; blue, staining score +2; orange, staining score +3.

**Figure 5.**
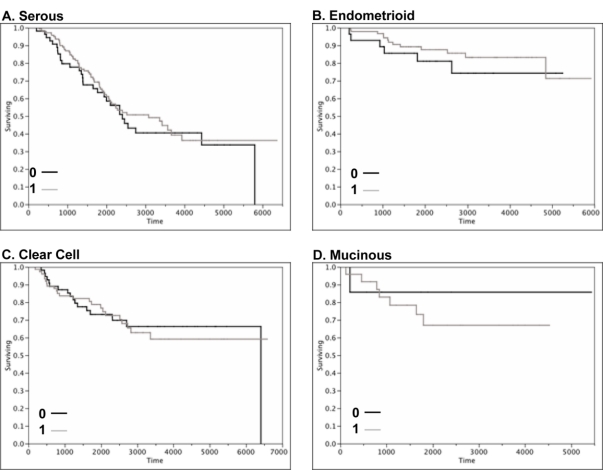
**Disease-specific survival by ovarian cancer subtype**. Kaplan-Meyer survival curve of TMA data for ovarian cancer patients shown by subtype. Disease-specific survival measured in days. Positive scores were binarized and are shown in gray. Black, claudin 4 negative.

As shown by Kaplan-Meier curves (Figure 4), there was no association between claudin 4 expression and disease-specific survival or relapse-free survival in ovarian cancer overall. When the survival data was examined by histological subtype (Figure 5), there was no significant association between claudin 4 expression and survival in any of the ovarian cancer subtypes. In a smaller cohort of 42 high grade serous tumors, Litkouhi *et al.* also found no association between claudin 4 expression and survival [37]. In contrast, Lanigan *et al.* recently reported that overexpression of claudin 4 was associated with an adverse outcome in breast cancer [30]. Similarly, claudin 4 overexpression is associated with poor outcome in clear cell renal cell carcinoma [42]. Although the results from the TMA studies did not provide an association between claudin 4 protein expression and survival, an important finding was made. Namely, the data shows that claudin 4 protein is expressed by the majority of ovarian cancer tissues, but not by normal surface epithelial cells of the ovary, and thus may serve as a target for therapy. The implications for the role of claudin 4 in other functional aspects of ovarian cancer dissemination were therefore pursued.

### 2.5. Claudin 4 Plays a Role in Spheroid Formation/Integrity

**Figure 6.**
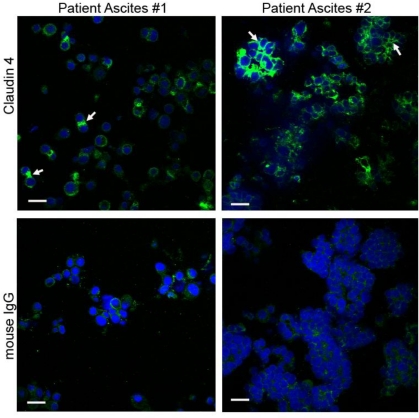
**Claudin 4 expression in patient spheroids**. Immunocytochemical staining of spheroids isolated from the ascites of two representative stage III/IV serous ovarian cancer patients. Spheroids were stained with either an antibody against claudin 4 or normal mouse IgG, followed by a FITC-conjugated secondary antibody (green). Nuclei were stained with DAPI (blue). Arrows indicate claudin 4 staining at sites of cell-cell contact. Bar = 20 μm.

Our finding that claudin 4 is localized primarily to the membrane in immunohisotchemically stained slides corresponds to previous reports by ourselves and others [13,15,37]. However, other studies have shown claudin 4 staining of both the cytoplasm and membrane in some serous ovarian cancer tumors, suggesting that in addition to its role in tight junction formation, claudin 4 may have additional functions regulating proliferation or differentiation [32,38]. In an effort to explore the potential functional role of claudin 4 in ovarian cancer dissemination and determine whether functional tight junctions are formed, we examined claudin 4 protein expression and localization by immunocytochemistry in cells from the ascites of ovarian cancer patients. Late stage ovarian cancers are frequently associated with the accumulation of peritoneal ascites fluid, which may contain ovarian cancer cells present either singly or as multicellular spheroids. Eight of 10 ascites samples showed positive claudin 4 membrane staining, either in single cells or multicellular aggregates (spheroids) or both, with strong staining visible at the points of cell-cell contact (Figure 6, arrows). Our findings are consistent with those of Kleinberg *et al.*, who observed claudin 4 staining in over 90% of 218 ovarian cancer effusions examined [43].

**Figure 7.**
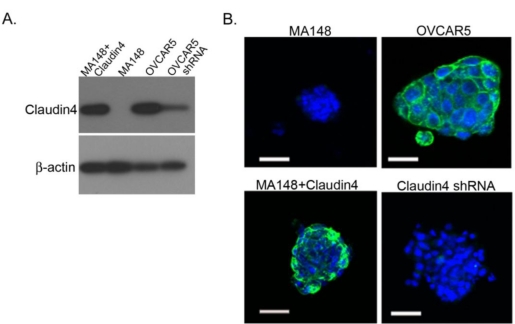
(**A**) Western blot showing claudin 4 expression levels in ovarian cancer cell lines engineered to express different levels of claudin 4 (10 μg protein/lane): MA148 cells transfected with claudin 4; MA148 cells transfected with an empty vector; NIH:OVCAR5 cells; and NIH:OVCAR5 cells treated with shRNA targeted to claudin 4. (**B**) *In vitro* cultured spheroids from cell lines engineered to express different levels of claudin 4. Spheroids were cultured for 48 hours, then stained with an antibody against claudin 4 followed by a FITC-conjugated secondary antibody (green). Nuclei were stained with DAPI (blue). Top left, MA148 cells transfected with an empty vector; bottom left, MA148 cells transfected with claudin 4; top right, NIH:OVCAR5 cells; and bottom right, NIH:OVCAR5 cells treated with shRNA targeted to claudin 4. Bar = 50 μm.

To determine whether the expression of claudin 4 may affect the formation of the spheroids which are frequently found in the ascites of ovarian cancer patients, we engineered two ovarian cancer cell lines to express different levels of claudin 4. The Western blot in Figure 7A shows no endogenous expression of claudin 4 in the MA148 cell line, while the NIH:OVCAR5 cell line expresses high levels of endogenous claudin 4. A claudin 4 transgene was then ectopically expressed in MA148 cells, and claudin 4 protein was shown to be expressed at high levels (Figure 7A). Conversely, NIH:OVCAR5 cells were transfected with an shRNA directed against claudin 4, resulting in a decrease in claudin 4 expression levels (Figure 7A).

Immunocytochemistry of spheroids formed *in vitro* from cultured cells expressing different levels of claudin 4 show claudin 4 localized to the membrane in cultured spheroids, similar to spheroids from patient ascites (Figure 7B). Although the NIH:OVCAR5 cells expressing an shRNA against claudin 4 have substantially reduced levels of claudin 4 protein, they were still able to form spheroids. Similarly, the ovarian cancer cell line MA148 had undetectable levels of claudin 4 expression, yet these cells formed compact multicellular spheroids *in vitro*; demonstrating that claudin 4 expression is not essential for spheroid formation. Previous analysis of breast and ovarian cancer cell lines suggests that a diverse array of adhesion molecules, including cadherins and beta 1 integrin, are involved in spheroid formation *in vitro,* depending on the cell line [8,44].

### 2.6. Claudin 4 Increases the Rate of Ovarian Cancer Spheroid Formation

**Figure 8.**
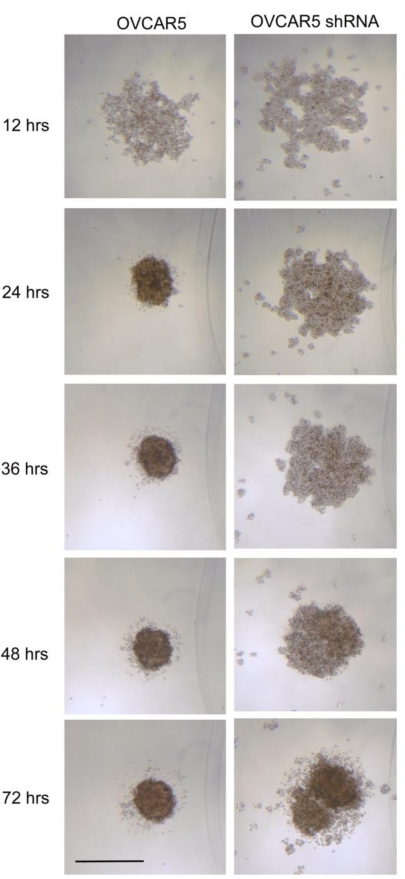
**Time course of spheroid formation in NIH:OVCAR5 cells**. Spheroids were formed *in vitro* using the liquid overlay method. Cells were grown in 96-well plates, with 2400 cells plated per well. Spheroid formation was observed at intervals over 72 hr. Experiments were repeated at least twice. Bar = 500 μm.

Although claudin 4 is not absolutely required for spheroid formation, we examined *in vitro* spheroid formation over time in ovarian cancer cells expressing different levels of claudin 4. For both ovarian cancer cell lines tested (NIH:OVCAR5 and MA148), tight, round multicellular aggregates or spheroids formed from single cells after approximately 24 hr. The size of the spheroids formed *in vitro* was at least partially dependent upon the number of cells plated (data not shown). As shown in Figure 8, the three-dimensional structures formed by NIH:OVCAR5 cells differed according to their expression of claudin 4 shortly after seeding. The parental NIH:OVCAR5 ovarian cancer cell line expressed high levels of claudin 4 and was able to form compact spheroids *in vitro* after 24 hr in culture, while the spheroids formed from NIH:OVCAR5 cells treated with shRNA targeting claudin 4 remained as loosely associated aggregates. The time required to form “true spheroids” (defined as tight round, regular, large, nonpermeable structures) increased in the absence of claudin 4 from about 24 hr for NIH:OVCAR5 cells to over 60 hr for NIH:OVCAR5 cells treated with shRNA targeting claudin 4. Although the differences in spheroid structure between NIH:OVCAR5 cells and NIH:OVCAR5 cells treated with shRNA diminished over time, the size of the spheroids after 72 hr suggests that increased levels of claudin 4 expression contribute to compact spheroid formation. In addition to the parental NIH:OVCAR5 cells, an empty vector control could also have served as a negative control; however, the Western blot in Figure 7A shows similar levels of beta actin in the parental and shRNA cells, suggesting that the shRNA does not have any generalized “off-target” effects in NIH:OVCAR5 cells.

Our analysis of the time to spheroid formation shows that claudin 4 expression contributes to compact spheroid structure. Sodek *et al.* previously showed that the formation of compact spheroids by ovarian cancer cells was associated with contractile behavior and an invasive phenotype [8]. Cancer cells grown as spheroids are also known to be chemoresistant, which is due, in part, to their structure [9]. Perhaps claudin 4 and tight junctions contribute to this by functioning as a barrier to chemotherapy. Alternatively, cell-cell adhesion could activate prosurvival signaling in spheroids [10,45–47]. Interestingly, claudin 4 was identified in a proteomic analysis of chemoresistance in ovarian cancer as one of 58 proteins that were overexpressed in cisplatin resistant cells [48].

The time required for MA148 cells to form spheroids was not dependent upon the presence of transfected claudin 4 (data not shown). The ability of MA148 cells to form spheroids in the absence of claudin 4 suggests that a redundant system of cell-cell adhesion may be used for spheroid formation. Other claudins, especially claudin 3, are also overexpressed in ovarian cancer and could be part of this redundancy [11,13–15,32,38,44,49].

### 2.7. Claudin 4 Decreases Paracellular Permeability

**Figure 9.**
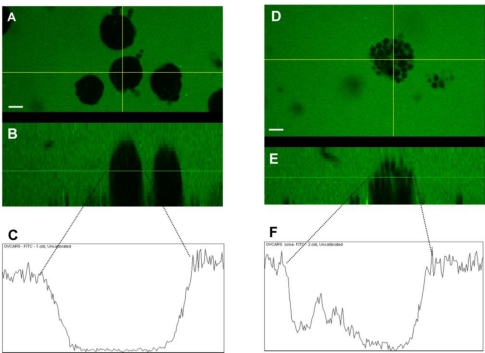
**Paracellular permeability of cultured spheroids expressing different levels of claudin 4.** (**A**) Photograph taken on fluorescent microscope *en face* of NIH:OVCAR5 spheroids incubated in FITC-dextran. (**D**) Photograph taken on fluorescent microscope *en face* of NIH:OVCAR5 spheroids treated with shRNA targeting claudin 4 incubated in FITC-dextran. Spheroids in (A) that express high levels of claudin 4 are tighter than those in (D) that express low levels of claudin 4. The fluorescence level of the perpendicular (Z) planes indicated by the horizontal yellow lines in (A) and (D) are shown in panels (B) and (E); providing a cross-sectional view of the tight aggregates (A, B) *vs.* the loose aggregates (D, E). Bar = 20 μm. Panels (C) and (F) show fluorescence profiles of spheroids in panels (B) and (E) as described in the Experimental section.

We also examined the paracellular permeability of spheroids expressing different levels of claudin 4 by testing the ability of spheroids formed *in vitro* to exclude FITC-dextran. At early time points (1–3 days), more compact spheroids capable of dye exclusion were formed in NIH:OVCAR5 cells compared to NIH:OVCAR5 cells treated with shRNA targeting claudin 4 (Figure 9); suggesting that claudin 4 levels are related to paracellular permeability and tight junction barrier function in spheroids. No difference in dye exclusion was observed at 8 days (data not shown). Differences in paracellular permeability between the empty vector and the claudin 4 transfected MA148 cells were more subtle. Slight differences in dye infiltration were observed, but overall the 3D structure and tightness of the spheroids did not appear to be affected (data not shown).

Paracellular resistance in ovarian cancer cell monolayers has been shown to be directly related to levels of claudin 4 expression [37]. Further studies have shown that phosphorylation of claudin 4 decreases the assembly of claudin 4 in tight junctions, thereby enhancing paracellular permeability [50,51]. In colon cancer cells, overexpression of claudin 4 decreased paracellular permeability and increased invasiveness [33]. Together with the observation that compact ovarian cancer spheroids are more invasive than diffuse spheroids [8], our results suggest that increased claudin 4 expression could be associated with invasiveness in ovarian cancer as well. Again, our TMA findings that claudin 4 protein was expressed by the majority of ovarian cancer tissues suggest that claudin 4 may serve as a target for therapy.In the past, spheroids have been shown to play an important role in ovarian cancer dissemination and invasion [5,6,8,52,53] and may also contribute to chemoresistance [9,54]. In this work, we showed that cells expressing high levels of claudin 4 were able to form compact spheroids more rapidly than cells with lower levels of claudin 4 expression, and paracellular permeability was increased in spheroids expressing reduced levels of claudin 4. These results suggest that claudin 4 may mediate chemoresistance in spheroids by increasing tight junction barrier function, and implicate claudin 4 as a target for therapy. The ability of the C-terminal fragment of the *Clostridium perfringens* enterotoxin (CPE), a polypeptide that causes food poisoning and binds to claudin 4 as a cellular receptor [55,56], to disrupt tight junction formation and increase paracellular permeability has been shown in embryogenesis [57] and ovarian cancer cell lines [37], and could potentially be used to increase the sensitivity of ovarian cancer cells to standard chemotherapy [58].

## 3. Experimental Section

### 3.1. Reagents

Cell culture media and supplements were purchased from Invitrogen Corporation (Carlsbad, CA) unless otherwise stated. Chemicals were purchased from Sigma-Aldrich (St. Louis, MO) unless otherwise stated.

Antibodies used were mouse anti-human claudin 4 (clone 3E2C1; Invitrogen), mouse anti-human β-actin (clone AC-74; Sigma-Aldrich), and normal mouse IgG (clone 3-5D1-C9; AbCam). Secondary antibodies used were FITC-conjugated goat anti-mouse IgG + IgM (Roche Diagnostics, Indianapolis, IN), stabilized horseradish peroxidase-conjugated goat anti-mouse IgG (Thermo Fisher Scientific, Rockford, IL), and biotinylated horse anti-mouse IgG (Vector Laboratories, Burlingame, CA).

### 3.2. Cell Lines

Ovarian cancer cell lines SKOV3, ES-2, NIH:OVCAR3, HEY, C-13, OV2008, OVCA429, OVCA433, A2780-S, and A2780-CP (provided by Dr. Barbara Vanderhyden, University of Ottawa, Canada), NIH:OVCAR5 (provided by Dr. Judah Folkman, Harvard Medical School, Boston, MA), CAOV3 (provided by Dr. Robert Bast Jr., University of Texas, Houston, TX), and MA148 (provided by Dr. Sundaram Ramakrishnan, University of Minnesota, Minneapolis, MN) were maintained as previously described [6,59–61]. SKOV3, ES-2, and OVCA429 cell lines were derived from clear cell carcinomas; OV2008 and C-13 cell lines were derived from endometrioid tumors; NIH:OVCAR3, NIH:OVCAR5, OVCA433, CAOV3, HEY, MA148, A2780-S, and A2780-CP cell lines were derived from serous adenocarcinomas [59,60].

Immortalized normal ovarian surface epithelial (NOSE) cell lines 1816–575, 1816–686, IMCC3, IMCC5, and HIO117 (provided by Dr. Patricia Kruk, University of South Florida, Tampa, FL), and IOSE-29 and IOSE-80 (provided by Dr. Nelly Auersperg, University of British Columbia, Vancouver, BC, Canada) were also maintained as described [62,63]. Cells were maintained in a humidified chamber at 37 °C with 5% CO_2_ and were routinely subcultured with trypsin/EDTA.

### 3.3. shRNA Knockdown of Claudin 4

NIH:OVCAR5 cells were stably transfected with shRNA clone TRCN0000116631 (Open Biosystems, Huntsville, AL) plasmid DNA using Lipofectamine 2000 (Invitrogen) according to the manufacturer’s instructions.

### 3.4. Transfection of MA148 Cells with Claudin 4

The claudin 4 coding sequence was amplified from CAOV3 total RNA using the Access one step RT-PCR kit (Promega, Madison, WI) with primers; Forward, AGATCTATGGCCTCCATGGGG; Reverse, TCTAGATTACACGTAGTTGCTGGCAGC, and cloned into the TA cloning vector pCR2.1 (Invitrogen) according to the manufacturer’s instructions. The claudin 4 coding fragment was excised from pCR2.1 by digestion with BglII and XbaI and ligated into the pcDNA3.1 expression vector (Invitrogen), and the sequence and orientation were verified by sequencing with vector primers. The pcDNA3.1-claudin 4 plasmid was transfected into MA148 cells using Lipofectamine 2000 (Invitrogen) according to the manufacturer’s instructions. Stable clones were selected with neomycin.

### 3.5. Tissue Samples

Snap-frozen tissue samples and formalin-fixed, paraffin-embedded (FFPE) tissue blocks were obtained from the University of Minnesota Tissue Procurement Facility (TPF) after IRB approval. Snap-frozen tissues were used for isolation of RNA and protein; FFPE tissues blocks were used to optimize immunohistochemical staining. The seven snap-frozen ovarian cancer tissues used for RNA and protein analysis were derived from the primary ovarian tumors of women with stage III/IV ovarian cancer of the serous subtype. The five snap-frozen normal ovarian tissues were obtained from patients with benign leiomyomas, endometriosis, benign peritubal cysts, or other non-ovarian diseases. For immunohistochemistry, 33 serous tumors, 4 clear cell tumors, and 21 normal ovaries were examined. All tissue samples underwent strict quality control measures prior to use in these studies. Namely, tumors were diagnosed by a pathologist at the time of surgery using OCT embedded tissue. The following day, the FFPE H&E slides were reviewed by a pathologist to confirm the accuracy of the diagnosis. A third pathologist reviewed the quality control H&E slides of all TPF cases to confirm the diagnosis of the samples prior to distribution to researchers. Additionally, a pathologist (S.E.P.) reviewed the slides while scoring the IHC staining.

### 3.6. Isolation of Spheroids from the Ascites of Ovarian Cancer Patients

Ascites fluid was obtained from the University of Minnesota Tissue Procurement Facility after IRB approval. Spheroids were isolated from ovarian cancer patient ascites as previously described [5]. Briefly, ascites was centrifuged at 500–700 × g for 10 min and erythrocytes were lysed by resuspending cells in lysis buffer (10 mM potassium bicarbonate, 155 mM ammonium chloride, 0.1 mM EDTA, pH 7.5) for 5 min. Remaining cells were collected by centrifugation, washed with PBS and viably frozen (10% dimethyl sulfoxide in fetal bovine serum) and stored in liquid nitrogen until use.

### 3.7. RNA Extraction and Reverse Transcriptase Polymerase Chain Reaction

Total RNA was extracted from cell lines and ovarian tissue samples using the RNeasy Mini kit (Qiagen, Valencia, CA) according to the manufacturer’s instructions. A 372 bp sequence corresponding to claudin 4 was amplified from 200 ng of total RNA using the following primers: Forward, 5′ TGATATCACCTCTGGGACTGT’; Reverse, 5′ CAGAAACCACAAAGAAGGAAG. One-step RT-PCR was performed with the RT-PCR Access kit (Promega, Madison, WI), with conditions as follows: 45 min at 45 °C; 1 cycle of 94 °C, 2 min; 56 °C, 1 min; 72 °C, 1 min; 35 cycles of: 94 °C, 30 sec; 56 °C, 1 min; 72 °C, 1 min; and a final extension at 72 °C for 7 min. Expression of β-actin in the samples confirmed that RNA was not degraded and that similar amounts of RNA were loaded [β-actin primers (Forward, 5′GGCCACGGCTGCTTC; Reverse, 5′GTTGGCGTACAGGTCTTTGC)]. These experiments were performed at least two times.

### 3.8. Quantitative Reverse Transcriptase Polymerase Chain Reaction

Real time quantification of claudin 4 was performed using the SYBR-green assay (Bio-Rad Laboratories, Hercules, CA) and the iQ5 Real-Time PCR thermocycler (Bio-Rad). Two micrograms of total RNA was used for cDNA synthesis using an oligo dT primer and Superscript III first-strand synthesis kit (Invitrogen) according to the manufacturer’s specifications. Two microliters of cDNA was amplified in a 25 μL reaction containing 1 μL each of claudin 4 forward and reverse primers (Forward, CTTCATCGGCAGCAACATT; Reverse, AGCAGCGAGTCGTACACCTT), and 13 μL of iQ SYBR green supermix (Bio-Rad). Following an initial denaturation step of 95 °C for 3 min, 40 cycles of PCR were performed under the following conditions: 95 °C, 10 sec (denaturation) and 52 °C, 30 sec (annealing/extension). All real-time PCR reactions were run in duplicate and melt curve analysis was performed to determine amplification of a single product. Data was normalized to the amount of β-actin present in the sample, determined in a separate reaction [primers β-actin forward: AGAGCTACGAGCTGCCTGAC; β-actin reverse: GGATGTCCACGTCACACTTC; and annealing temperature 54 °C]. Transcript levels were quantitated using cRNA standard curves for claudin-4 and β-actin [64] and the relative amount of each sample was determined as a fold-change increase over the lowest expressing cell line (1816–575). Expression values reported in Figure 1A are the average of two experiments, except samples IMCC3, IMCC5, HIO117 and IOSE-29 which were run in duplicate in a single experiment.

### 3.9. Western Immunoblotting

Protein was extracted from snap-frozen tissues in T-PER (Tissue Protein Extraction Reagent; Thermo Fisher Scientific) containing a protease inhibitor cocktail (Roche Applied Science, Basel, Switzerland). Total protein extracts were also derived from confluent monolayers of cells in 50 mM Tris, 150 mM sodium chloride, 1 mM EDTA, 1% Triton X-100, 1% sodium deoxycholate, 0.1% SDS, protease inhibitor cocktail (Roche Applied Science), and 1 mM PMSF then stored at −80 °C. Protein concentration was determined using the BCA Protein Assay (Thermo Fisher Scientific). Fifty micrograms of protein were separated on a 10% SDS Tris-HCl polyacrylamide gel or a 4–20% Tris-HCl Criterion precast gel (BioRad), then blotted onto a polyvinylidene difluoride membrane (GE Healthcare Limited; Piscataway, NJ). Membranes were blocked with 5% powdered milk (Roundy’s Inc.; Milwaukee, WI) in PBS as previously described [65,66], and then incubated in 0.167 μg/mL mouse anti-human claudin 4 (clone 3E2C1) overnight, followed by a 2-hr incubation in horseradish peroxidase conjugated goat anti-mouse antibody diluted 1/5000. Protein was visualized using the Super Signal West Femto kit (Thermo Fisher Scientific) according to manufacturer’s instructions. Membranes were exposed to autoradiography film (Midwest Scientific; Valley Park, MO) and developed. Blots were reprobed with an antibody against β-actin as a loading control.

### 3.10. Immunohistochemical Staining of Tissues

FFPE tissue sections were deparaffinized and rehydrated through a series of xylene and ethanol washes as previously described [66]. Antigen retrieval was performed in a citrate buffer (Biocare, Concord, CA) and endogenous peroxidase activity was blocked with hydrogen peroxide. Slides were incubated with mouse anti-human claudin 4 monoclonal antibody (clone 3E2C1; Invitrogen) or normal mouse IgG1 (clone 3-5D1-C9; AbCam) at 0.25 μg/mL overnight. Slides were washed and incubated with biotinylated horse anti-mouse IgG (Vector Laboratories, Burlingame, CA), followed with an avidin:biotin complex (Vector Laboratories). Staining was visualized with 3,3′-diaminobenzidine (Biocare). Slides were examined by a pathologist (S.E.P.) in a blinded manner and assigned a score of 0 (no staining); 1 (<10% of neoplastic cells staining); 2 (10–50% of neoplastic cells staining); or 3 (>50% of neoplastic cells staining). FFPE blocks of human intestine were used as a positive control for claudin 4 antibody staining.

### 3.11. Tissue Microarrays

TMA slides containing 0.6 mm duplicate core samples for 500 ovarian cancer patients were provided by the Cheryl Brown Ovarian Cancer Outcomes Unit (University of British Columbia; Vancouver, BC, Canada). Patients included in the TMA were chosen based on having been optimally cytoreduced at initial surgery with no macroscopic residual disease remaining. Due to these criteria, a significant proportion of early stage cases were present on the TMA relative to the general population of patients with ovarian cancer. None of the patients received neoadjuvant therapy and all received platinum-based chemotherapy following surgery. The 500 cases included on the TMA were collected up to 18 years prior to this analysis. Hematoxylin and eosin stained slides for all cases were reviewed by a gynecologic pathologist (C.B.G.) to confirm diagnosis, stage, tumor cell type, and grade prior to TMA inclusion to ensure that the current diagnostic criteria for subclassification of ovarian cancer based on cell type were uniformly applied [67,68]. Samples displaying multiple cell types (mixed tumors) were excluded from the study. Details regarding the cohort used for these TMAs are provided in Table 1 and in Gilks *et al.* [40]. Patients were followed for a median of 4.6 (0.1–18) years after the initial surgery. Three-tiered grading of ovarian cancer tissues was done using the Silverberg grading system at the time of review of the complete slide sets for all cases on the ovarian TMAs [69].

Tissue microarray slides were treated and stained identically to the individual tissue sections, and scored in a blinded manner as described above. In cases where the duplicate core samples received different scores, results were averaged for analysis. For some analyses, scores of 1, 2, and 3 were grouped and considered positive (“binarized data”).

### 3.12. TMA Statistical Analysis

Differential expression for claudin 4 across the histopathological subtypes was assessed with the Pearson Chi-Square statistic. Univariable relapse-free survival for the entire cohort and each histopathologic subtype was examined with Kaplan-Meier survival curves. Results significant in univariable analysis were subjected to multivariable relapse-free survival using the Cox Proportional Hazards test. The level of significance for all comparisons was p < 0.05. All statistical calculations were computed with JMP v. 6.0.3 (SAS Institute Inc., Carey, NC).

### 3.13. Spheroid Formation *in Vitro*

Spheroids were cultured using the liquid overlay method, as previously described [52]. Briefly, 96-well tissue culture plates were coated with 100 μL of 0.5% w/v SeaKem LE agarose (Lonza, Walkersville, MD) in serum-free culture media, to prohibit cell adhesion to the substratum. Plates were allowed to cool for at least 30 min at room temperature. Cells grown in monolayer cultures were released with 0.5% trypsin, 2 mM ethylenediaminetetraacetic acid (Invitrogen) and resuspended in complete cell culture media. The cell suspension was run through a 70 μm cell strainer (BD Biosciences, Bedford, MA) to remove residual clumps. Cells were counted with a hemocytometer, then diluted to 2000 to 170,000 cells/mL. Cell suspensions were layered on top of the agarose-coated plates at a volume of 100 μL/well and then incubated at 37 °C.

### 3.14. Immunocytochemical Staining of Spheroids

Spheroids either isolated from the ascites of ovarian cancer patients or formed *in vitro* from ovarian cancer cell lines were embedded in thrombin clots and 20 μm OCT-frozen sections were stained as previously described [52]. Alternatively, spheroids were fixed and stained in a 96-well plate prior to mounting onto slides. Briefly, spheroids were washed three times with Dulbecco’s phosphate buffered saline (DPBS) containing calcium and magnesium, and centrifuged at ∼200 × g for 5 min. Cells were fixed in 200 μL of ice cold 100% methanol overnight at −20 °C, then rehydrated with 3 washes of DPBS containing calcium and magnesium. Cells were blocked with 1% normal goat serum, 0.3% Tween-20 in DPBS containing calcium and magnesium for 1 hr, then incubated in 100 μL of primary antibody at 2.5 μg/mL in blocking buffer overnight at 4 °C with gentle agitation. Cells were washed three times in blocking buffer and incubated in a 1:100 dilution of secondary antibody (FITC-conjugated goat anti-mouse IgG + IgM; Roche Diagnostics) overnight at 4 °C in the dark. Cells were washed three times in blocking buffer and incubated in 100 μL of a 2.86 × 10^−7^ M DAPI solution for 5 min, then washed three times in DPBS containing calcium, magnesium, and 0.3% Tween-20 with a final wash in SlowFade equilibration buffer (Invitrogen, Eugene, OR). Cells were mounted in 1× SlowFade reagent in PBS containing 50% glycerol.

### 3.15. FITC-Dextran Paracellular Permeability Imaging

Spheroids were formed in 6-well plates by coating plates with 2 mL agarose as described above. Cells were plated in 2 mL complete media at concentrations from 10,000 to 500,000 cells per well and spheroids were formed for 48 to 96 hr at 37 °C. Thirty minutes prior to visualization, a 4 kDa conjugate FITC-dextran (Sigma-Aldrich) was added to a final concentration of 0.05–0.1% [33,51]. Spheroids were imaged *in situ* on an Olympus FluoView FV1000 upright confocal microscope with a 20X water immersion objective and 488 nm laser. Fluorescence intensity profiles were generated with Image J 1.37v software.

### 4. Conclusions

In this study, we validated our previous gene microarray data, showing that claudin 4 RNA and protein is overexpressed in ovarian cancer tissues and cell lines compared to tissues and cell lines from normal ovaries. We also demonstrated that claudin 4 is differentially expressed across histological subtypes of ovarian cancer; however, no difference in survival was observed between claudin 4 positive *vs.* negative tumors. Claudin 4 was also expressed in ovarian cancer spheroids isolated from the ascites of patients. The parental NIH:OVCAR5 ovarian cancer cell line expressed high levels of claudin 4 and was able to form compact spheroids *in vitro* more rapidly than when the cell line was treated with shRNA targeting claudin 4, causing low levels of claudin 4 to be expressed. These results demonstrate a role for claudin 4 in spheroid formation and integrity, and lead us to speculate that claudin 4 may play a role in mediating chemoresistance in spheroids by increasing tight junction barrier function or activation of prosurvival signaling. Furthermore, as the majority of cases of ovarian cancer examined exhibited elevated levels of claudin 4 protein expression, this supports the use of claudin 4 as a therapeutic target, and we postulate that blocking claudin 4 function may increase the efficacy of chemotherapy delivered intraperitoneally. Indeed, several groups have reported using claudin 4 as a target for delivery of toxins and fluorescent molecules to ovarian and breast cancer cells [70–72].

## Figures and Tables

**Table 1.** Subtype, stage, Silverberg grade, and Claudin 4 score of ovarian cancer tissue microarrays.

**Subtype**	**Median Age (range)**	**Stage**	**N**	**Claudin 4 Positive (%)**	**Silverberg Grade**	**N**	**Claudin 4 Positive (%)**	**Claudin 4 Positive Overall (%)**
Serous (n = 212)	59.6 (33.5–86.0)	I	50	33 (66.0%)	1	12	7 (58.3 %)	153 (72.2 %)
		II	93	69 (74.2%)	2	56	37 (66.1%)	
		III	69	51 (73.9%)	3	144	109 (75.7%)	

Endometrioid (n = 125)	54.1 (29.4–88.1)	I	69	54 (78.3%)	1	82	66 (81.5%)	96 (77.4 %)
		II	50	37 (75.5%)	2	35	27 (77.1%)	
		III	6	5 (83.3%)	3	8	3 (37.5%)	

Clear Cell (n = 132)	55.0 (28.1–89.0)	I	68	45 (66.3%)	1	0*	N/A	76 (57.6 %)
		II	56	26 (46.4%)	2	0*	N/A	
		III	8	5 (62.5%)	3	132	76 (57.6 %)	

Mucinous (n = 31)	56.4 (25.4–76.7)	I	18	15 (83.3%)	1	11	8 (72.7%)	24 (77.4%)
		II	12	8 (66.7%)	2	18	14 (77.8%)	
		III	1	1 (100%)	3	2	2 (100.0%)	

Total (n = 500)	56.6 (25.4–89.0)	I	205	147 (71.7%)	1	105	81 (77.1%)	349 (69.9%)
		II	211	140 (66.4%)	2	109	78 (71.6%)	
		III	84	62 (73.8%)	3	286	190 (66.4%)	

* All clear cell carcinomas are considered high grade.
